# IgG4-related disease in gastroenterology: from pathogenesis to clinical management and long-term outcomes

**DOI:** 10.1007/s00535-026-02424-2

**Published:** 2026-05-03

**Authors:** Yifei Wang, Luyi Peng, Wen Zhang

**Affiliations:** Department of Rheumatology, Peking Union Medical College Hospital (PUMCH), Chinese Academy of Medical Sciences and Peking Union Medical College, National Clinical Research Center for Rheumatic and Autoimmune Diseases (NCRC-RAD), State Key Laboratory of Complex Severe and Rare Diseases, Key Laboratory of Rheumatology and Clinical Immunology, Ministry of Education, 1 Shuai-Fu-Yuan, Dongcheng District, Beijing, 100730 China

**Keywords:** IgG4-related disease, Autoimmune pancreatitis, IgG4-related sclerosing cholangitis, Fibroinflammatory disease

## Abstract

IgG4-related disease is a rare, immune-mediated, multisystem fibroinflammatory condition. It is characterized by elevated serum IgG4 concentrations and tissue infiltration of IgG4-positive plasma cells with distinctive histopathological features, including storiform fibrosis and obliterative phlebitis. Gastroenterological involvement is diverse and represents a critical diagnostic consideration, encompassing the pancreas, bile ducts, liver, esophagus, stomach, and intestine. In this review, we summarize the current understanding of genetic susceptibility and environmental risk factors underlying this disease, and systematically describe the clinical presentations, histopathological characteristics, imaging findings, and serological profiles of each major gastroenterological manifestation. We further review evidence-based treatment regimens ranging from glucocorticoids and conventional immunosuppressants to emerging biologic therapies, discuss organ-specific therapeutic responses, and identify predictors of disease relapse along with long-term surveillance strategies. From a gastroenterologist’s perspective, this review aims to provide a practical and integrated framework to facilitate early recognition, accurate differentiation from mimicking conditions such as pancreaticobiliary malignancies, and optimized long-term management of this complex yet treatable disease.

## Introduction

IgG4-related disease (IgG4-RD) is a chronic, immune-mediated fibroinflammatory condition that can affect virtually any organ system. It is histopathologically characterized by three hallmark features: dense lymphoplasmacytic infiltration, storiform fibrosis, and obliterative phlebitis, typically accompanied by abundant IgG4-positive plasma cells and elevated serum IgG4 concentrations [1].

Although individual organ manifestations now recognized as part of IgG4-RD, such as Mikulicz disease, were described over a century ago, the concept of IgG4-RD as a unified systemic entity only emerged in the early 2000s [2]. This paradigm shift was catalyzed by two pivotal discoveries: first, the 2001 report by Hamano et al. linking elevated serum IgG4 to sclerosing pancreatitis [3], and subsequently, Kamisawa’s 2003 proposal of a systemic “IgG4-related autoimmune disease” after identifying analogous IgG4-positive plasma cell infiltration across multiple organs [4]. While pancreas, bile ducts, salivary glands, lacrimal glands, kidneys, and retroperitoneum are most frequently involved—in an international cohort of 493 patients, pancreato-hepatobiliary involvement was most common (47.7%), followed by salivary glands (37.7%), lacrimal glands (26%), retroperitoneum (15.8%), and kidneys (15.6%) [5]—organ distribution varies notably by race, with Asian patients showing higher rates of salivary and lacrimal gland involvement, while non-Asian patients more commonly present with pancreato-hepatobiliary disease [5]. The clinical spectrum of IgG4-RD continues to expand [6].

The estimated incidence of IgG4-RD ranges from 0.28 to 1.08 per 100,000 person-years in Japan to 1.39 per 100,000 person-years in the USA [7, 8]. The apparent increase in reported incidence in recent years is primarily attributed to greater clinical awareness and more refined diagnostic criteria, rather than a genuine rise in occurrence. The disease exhibits a striking male predominance and typically presents in middle-aged or elderly individuals, with a peak onset in the fifth to seventh decades of life [9, 10]. Notably, reported epidemiological variations across different ethnic groups underscore the potential roles of genetic susceptibility and environmental factors in disease pathogenesis.

For gastroenterologists, IgG4-RD presents critical diagnostic and management challenges, primarily manifesting as autoimmune pancreatitis (AIP) or IgG4-related sclerosing cholangitis (IgG4-SC) [10]. Accurate diagnosis hinges on integrating clinical presentation, serology, characteristic imaging findings, and the definitive histopathological triad [11, 12]. Despite an excellent initial response to corticosteroid therapy, management is complicated by high relapse rates (34%–58%) and a reported association with malignancy, making early recognition and vigilant long-term follow-up critical for optimizing patient outcomes [13, 14].

Here, we provide a comprehensive overview of IgG4-RD from a gastroenterological perspective, structured around four critical domains. We begin by examining genetic susceptibility and environmental risk factors that predispose to disease development. Subsequently, we systematically characterize the clinical, histopathological, imaging, and serological features of IgG4-RD across major gastroenterological organs. We then review evidence-based treatment regimens, including organ-specific therapeutic responses, and strategies for achieving remission. Finally, we identify predictors of disease relapse and outline surveillance strategies to optimize long-term outcomes. By integrating current diagnostic and therapeutic evidence, this review seeks to enhance clinical recognition and improve patient management in this complex yet treatable condition.

## Genetic susceptibility and risk factors

### Genetic susceptibility

Genetic predisposition in IgG4-RD exhibits both polygenic and rare monogenic inheritance patterns. A family history of autoimmune diseases was identified in 14.8% of IgG4-RD patients in a Chinese cohort, with IgG4-RD accounting for 17.2% of these familial autoimmune conditions [15]. Patients with a family history of autoimmune diseases demonstrated earlier disease onset and higher ANA positivity rates, indicating potential shared genetic predisposition with other autoimmune conditions [15].

HLA-DRB1 represents the strongest susceptibility locus for IgG4-RD [16], with population-specific associations: DRB1*04:05–DQB1*04:01 in Japanese populations [17], DRB1*16 in Europeans [16], and multiple independent HLA alleles in Chinese Han populations [18]. A 2025 whole-genome sequencing study in 869 Japanese patients expanded upon previous GWAS findings by identifying two additional independent HLA associations beyond the reported HLA-DRB1-GB-7 amino acid residue: HLA-A-GA2-9 and HLA-DQB1-GB-82, all located within peptide-binding grooves critical for antigen presentation [19]. Concordantly, a recent GWAS in 1115 Chinese patients identified 16 genetic susceptibility loci, with the strongest signal also mapping to the extended MHC region on chromosome 6 [18].

Beyond HLA, several loci involved in B-cell regulation have been implicated. FCGR2B, encoding the inhibitory Fc receptor IIB, represents the second genome-wide significant locus [16]. Whole-genome sequencing identified a novel intronic FCGR2B variant, rs13376485, not captured by previous microarray panels, with additional FC-γ receptor gene family loci identified on chromosome 1 in Chinese cohorts [18]. Additional non-HLA candidates include CTLA4 [20], FCRL3 [21], and IL1R1 variants [22], which show organ-specific associations.

Whole-genome sequencing also enabled direct assessment of complement component 4 (C4) copy number variations, revealing opposing effects of C4A and C4B isotypes on disease susceptibility. Lower C4A copy number conferred protective effects, while higher C4B copy number increased disease risk [19].

Subtype-specific analysis identified two novel susceptibility loci unique to Mikulicz’s disease: PTCH1 (rs66917126), encoding a sonic hedgehog pathway receptor implicated in embryonic development and tumorigenesis, and long non-coding RNA LOC102724227 (rs894063168). These organ-specific genetic determinants suggest distinct pathogenic mechanisms underlying clinical heterogeneity in IgG4-RD subtypes.

Recent genetic studies further identified monogenic IKZF1 and UBR4 variants in familial clusters, revealing FYN kinase-driven Th2 polarization as a key mechanistic pathway [23]. These findings demonstrate a shared genetic architecture with systemic lupus erythematosus, rheumatoid arthritis, and inflammatory bowel disease through HLA-mediated antigen presentation, B-cell regulatory dysfunction, and complement system dysregulation [16], explaining a reported comorbidity rate of approximately 10% with autoimmune rheumatic diseases [24]. Notably, IgG4-RD and systemic lupus erythematosus exhibit opposing genetic effects for shared susceptibility genes (FCGR2B, C4A, C4B, HLA-DRB1-GB-7), underscoring the complex interplay between genetic variants and immune phenotypes in autoimmune pathogenesis.

### Etiological and environmental risk factors

The etiology of IgG4-RD remains incompletely understood, with both genetic predisposition and environmental triggers contributing to disease development. Based on clinical observations and characteristics of IgG4 molecules, investigators have explored potential environmental risk factors, particularly occupational exposures.

De Buy Wenniger et al. first explored occupational risk factors for IgG4-RD. In their study, compared to the control cohort, among which 14% reported a history of blue-collar profession, 88% of IgG4-RD patients had worked in such professions throughout their careers [25]. Subsequent study replication found that 61% of patients had a blue-collar profession and recalled exposure to potentially harmful compounds; 52% reported intensive exposure to solvents, industrial dusts, or industrial compounds, while the control cohort reported 7% to 22% of relevant exposures.

Hubers et al. performed a matched case–control study in the Netherlands. Results showed that individuals with at least one year of blue-collar experience had higher odds of developing IgG4-RD than those who did not [26]. Furthermore, prolonged exposure to occupational hazards such as biological dusts, mineral dusts, gas fumes, vapors, pesticides, asbestos, chromium, diesel motor exhaust, nickel resulted in greater risks of developing IgG4-RD [26, 27]. Several small case reports also identified asbestos exposure, tobacco smoking, biomass fuel and COVID-19 infection as potential risk factors [28–32].

**Fig. 1:**
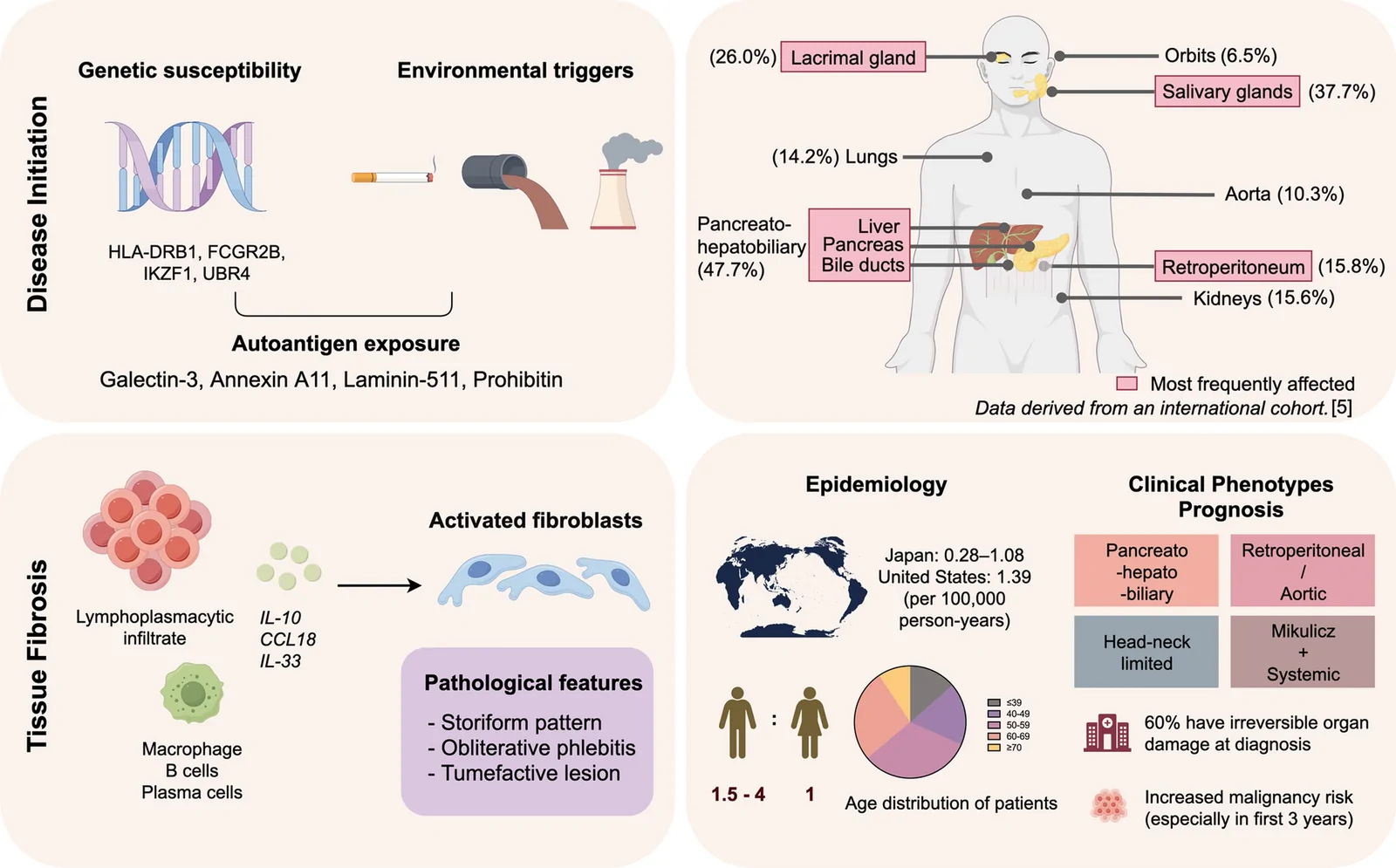
Pathogenesis, clinical spectrum, and epidemiology of IgG4-RD

Figure 1 summarizes the genetic susceptibility, environmental triggers, and immunological pathogenesis of IgG4-RD, along with the major organ systems affected and key epidemiological features.

## Major gastroenterology organ involvement

Table 1 provides a comparative overview of the clinical, histological, imaging, and serologic features across major gastroenterological manifestations of IgG4-RD.

**Table 1:** Comparative clinical features of major gastroenterological manifestations of IgG4-related disease

	AIP-1	IgG4-SC	Hepatic Involvement		Esophagus, Stomach, and Intestine Involvement
			IgG4-AIH	IPT	
Clinical Presentation	Jaundice, abdominal pain, steatorrhea, postprandial bloating, and weight loss	Obstructive jaundice, accompanied by pruritus, abdominal pain, and weight loss	Fatigue, lethargy, malaise, anorexia, and nausea; some may present profound jaundice	Abdominal pain, fever, weight loss, and malaise; but can be asymptomatic	Asymptomatic, dysphagia, odynophagia, anorexia, nausea, abdominal pain, weight loss, etc
Common histological finding: dense lymphoplasmacytic infiltrate, fibrosis arranged at least focally in a storiform pattern, obliterative phlebitis					
Histology	Lymphoplasmacytic Sclerosing Pancreatitis (LPSP): dense lymphoplasmacytic infiltrate, storiform fibrosis, and obliterative phlebitis	Consistent with general IgG4-RD histological features: Greater than 10 IgG4 + plasma cells per HPF in biopsy specimens, greater than 50 IgG4+ plasma cells per HPF in resection specimens, and an IgG4+ /IgG+ ratio exceeding 0.4	Interface hepatitis, infiltration of inflammatory cells, predominantly mononuclear cells	Myofibroblastic spindle cells with diffuse lymphoplasmacytic infiltration, high eosinophil and IgG4-positive plasma cell presence	Elevated number of IgG4 positive plasma cells, elevated IgG4: IgG ratio, characteristic pathological features
Imaging	CT: Diffuse sausage-like pancreas with hypoattenuating halo Enhanced CT: delayed enhancement MRI: T1 hypointense, T2 hyperintense lesions with restricted diffusion	Segmental long strictures with pre-stenotic dilation Ultrasound: circular symmetrical bile duct wall thickening with smooth margins, homogeneous internal echo, and preserved triple-layer stratification	No characteristic radiological findings have been reported, but liver fibrosis can be assessed by magnetic resonance and ultrasound-based elastography	CT: irregular and lobulated hypoattenuating lesion Enhanced CT: central hypoattenuating areas with delayed hyperattenuating periphery	Endoscopy can reveal esophagus stricture, esophageal mass, gastric ulcer, gastric lesions, intestinal polyps while radiological examinations may discover mass-like lesions
Serology	Elevated serum IgG4 level, hypergammaglobulinemia, peripheral eosinophilia, and increased serum IgE	Elevated serum alkaline phosphatase in most patients, elevated serum IgG4 levels	Increased concentrations of IgG and high ANA titer, elevated liver enzyme (including ALT, AST, ALP, γ-GTP)and presence of typical autoantibodies	Leukocytosis, elevated erythrocyte sedimentation rate, high CRP value, liver enzyme abnormality in some cases, negative for alpha-fetoprotein	Usually not remarkable, but elevated level IgG4 and liver enzymes may be present

### Type 1 autoimmune pancreatitis

Type 1 autoimmune pancreatitis (AIP-1) is the primary gastrointestinal manifestation of IgG4-RD [33]. Epidemiologically, patient demographics show geographic variation. The median age at presentation is 60–65 years in Asian cohorts, compared to 57 years in European populations [34]. Clinically, symptomatic presentation occurs in approximately 63% of patients [35]. Among symptomatic cases, obstructive jaundice is the predominant initial symptom, affecting approximately 75% of AIP-1 patients in international cohorts [36] and has a particularly high prevalence in Asian populations [37]. Abdominal pain is another frequent presentation [38]. Patients may develop acute pancreatic insufficiency at presentation with steatorrhea, postprandial bloating, increased stool frequency, and weight loss [36]. Long-term follow-up reveals additional complications including pancreatic atrophy, new-onset diabetes mellitus, and calcification formation [39].

Notably, over one-third of AIP-1 cases are identified incidentally in asymptomatic patients through imaging performed for unrelated indications [35]. Unlike other forms of pancreatitis, acute episodes are rare in AIP-1 [40].

The histopathological features of AIP-1, also referred to as lymphoplasmacytic sclerosing pancreatitis (LPSP), include a dense lymphoplasmacytic infiltrate, storiform fibrosis, and obliterative phlebitis, which align with the classical features of IgG4-RD [41].

As a pancreatic manifestation of IgG4-RD, AIP-1 exhibits distinctive imaging features that facilitate diagnosis and differentiation from pancreatic malignancy. Computed tomography (CT) imaging typically demonstrates diffuse sausage-like pancreatic enlargement with loss of lobulations and a hypoattenuating peripancreatic halo, showing delayed enhancement [42, 43]. Furthermore, focal (26%) and multifocal (14%) presentations also occur, most commonly involving the pancreatic head/neck, and may manifest as discrete masses [44]. Hallmark findings include main pancreatic duct strictures involving more than one‑third of the ductal length or presenting as multifocal narrowings [42, 45, 46]. Magnetic resonance imaging (MRI) reveals T1-hypointense and T2-hyperintense lesions with restricted diffusion. Magnetic resonance cholangiopancreatography characteristically shows the "duct-penetrating sign", indicating intact ductal traversal without obstructive changes [43]. Endoscopic ultrasound may reveal diffuse pancreatic enlargement with altered echotexture or focal hypoechoic masses, while fluorodeoxyglucose positron emission tomography/computed tomography displays intense uptake at pancreatic and extrapancreatic sites [45].

Key laboratory abnormalities include elevated serum IgG4 level (with higher positivity in Asian populations at 84–86% [35]), hypergammaglobulinemia [47], peripheral eosinophilia, and increased serum IgE. These serological changes aid diagnosis but require correlation with imaging and histology.

### IgG4-related sclerosing cholangitis

IgG4-related sclerosing cholangitis (IgG4-SC) is the principal hepatobiliary manifestation of IgG4-RD. It is reported in approximately 50–70% of patients with AIP-1, though isolated biliary involvement without pancreatic disease occurs in 10% of cases [48]. IgG4-SC predominantly affects elderly males in their sixth to eighth decades [49], typically presenting with obstructive jaundice accompanied by pruritus, abdominal discomfort, and weight loss [50]. Concurrent biliary and pancreatic involvement is highly suggestive of IgG4-RD.

According to the 2012 Japanese diagnostic criteria [51], IgG4-SC is classified into four cholangiographic types based on stricture distribution: Type 1 (64%) with isolated lower common bile duct stenosis, Type 2 (13%) with diffuse intrahepatic and extrahepatic involvement (subdivided into types 2a and 2b based on presence or absence of prestenotic dilation), Type 3 (10%) with both hilar and lower bile duct strictures, and Type 4 (10%) with isolated hilar stenosis. Imaging characteristically reveals segmental long strictures with pre-stenotic dilation [52, 53]. Endosonography, including endoscopic ultrasound and intraductal ultrasound, demonstrates circular symmetrical bile duct wall thickening with smooth margins, homogeneous internal echo, and preserved triple-layer stratification [54]. Distinctively, its wall thickening extends beyond strictured segments, distinguishing IgG4-SC from both primary sclerosing cholangitis (PSC) and cholangiocarcinoma [55, 56].

Histopathological confirmation remains essential for definitive differentiation. The hallmarks of histology in IgG4-SC are consistent with those of IgG4-RD. Distinguishing IgG4-SC from PSC and cholangiocarcinoma requires quantification of IgG4 + plasma cells and the IgG4 + /IgG + plasma cell ratio per high-power field (HPF) [57]. European diagnostic guidelines specify the following criteria [58]: greater than 10 IgG4 + plasma cells per HPF in biopsy specimens, greater than 50 IgG4 + plasma cells per HPF in resection specimens, and an IgG4 + /IgG + ratio exceeding 0.4.

Differential diagnosis between IgG4-SC and PSC can be challenging due to overlapping clinical and imaging features. A validated scoring system, the Kim score, incorporating three variables—patient age, presence of other organ involvement, and absence of beaded appearance on cholangiography—demonstrates excellent discrimination (AUC 0.986). Scores of 7–9 points indicate probable IgG4-SC, 5–6 points warrant a diagnostic steroid trial, while 0–4 points suggest probable PSC [59].

Laboratory tests typically reveal elevated serum alkaline phosphatase in most patients. Elevated serum IgG4 levels serve as key diagnostic markers alongside other liver function abnormalities. Recent research has uncovered several autoantigens associated with disease pathogenesis, such as annexin A11 [60], laminin 511-E8 [61], galectin-3 [62], and prohibitin 1 [62].

### Hepatic involvement

Hepatic involvement of IgG4-RD manifests in two forms, autoimmune hepatitis (AIH) and hepatic inflammatory pseudotumor (IPT) [63]. Histological analysis showed that hepatic IPT tissue merged seamlessly with inflammatory changes of bile ducts [64]. Furthermore, both forms of hepatic presentations can occur concomitantly with chronic cholangitis, raising the question of whether they represent distinct disease entities or sequelae of cholangitis [64–67].

#### IgG4-related autoimmune hepatitis

IgG4-related autoimmune hepatitis (IgG4-AIH) is defined as a disorder satisfying diagnostic criteria for both IgG-RD and AIH, an inflammatory disease caused by an autoimmune response against hepatocytes [68, 69]. Classical AIH exhibits a broad clinical spectrum from asymptomatic disease to fulminant hepatitis [64, 70–72]. AIH is serologically characterized by elevated serum IgG and autoantibodies: ANA and/or SMA in type 1; anti-LKM1 in type 2 [68, 73]; and is histologically defined by interface hepatitis with dense mononuclear cell infiltration at the portal area and the hepatic parenchyma [68, 69].

IgG4-AIH shares many characteristics with classical AIH. A systematic review summarizing 43 studies confirmed that most IgG4-AIH patients presented with elevated liver enzymes and serum IgG4 levels [74]. Most studies utilized > 10 IgG4-positive plasma cells per high-power field (HPF) as diagnostic criteria. Antibody profiling further revealed a high prevalence of positive ANA and SMA antibodies, whereas positivity for LKM antibodies and anti-mitochondrial antibodies (AMA) was notably rare [74], suggesting that IgG4-AIH predominantly overlaps with type 1 AIH. Most patients with IgG4-AIH can be identified based on the satisfaction of the IAIHG (International Autoimmune Hepatitis Group) scoring criteria, serum IgG4 concentration, and IgG4-bearing plasma cell infiltration (Table 1) [75].

Liver biopsy remains essential for diagnosis, as it helps to rule out chronic viral hepatitis, metabolic disease, and other overlap syndromes [76, 77]. Once confirmed, liver biopsy also becomes the basis for grading and staging of disease severity [76].

#### Hepatic inflammatory pseudotumor

IPTs are lesions consisting predominantly of myofibroblastic spindle cells with variable inflammatory cellular components, including plasma cells, lymphocytes, and macrophages, and sometimes containing areas of fibrosis and necrosis [78–81]. Based on histological composition, IPT can be classified into two types: fibrohistiocytic and lymphoplasmacytic [82]. The fibrohistiocytic subtype is characterized by xanthogranuloma and fibrosis with neutrophilic and multinucleated giant cell infiltration. Although this subtype can occasionally be enriched with IgG4-positive plasma cells, it is considered to represent a more heterogeneous group of disorders that histopathologically differ from IgG4-RD [82]. The lymphoplasmacytic subtype mainly contains diffuse lymphoplasmacytic infiltration with higher eosinophil and IgG4-positive plasma cell presence, and is thus more closely associated with IgG4-RD [80, 82, 83].

Clinically, patients often present with abdominal pain, fever, weight loss, and malaise, but they can also be asymptomatic, identifiable only through radiological findings and serological abnormalities [78, 79, 81, 84]. The two subtypes differ in their clinical profiles: patients with fibrohistiocytic IPT usually report subjective symptoms such as pain or fever, while patients with lymphoplasmacytic IPT seldom present with symptoms but are instead identified through abnormal laboratory findings reflecting liver dysfunction [82]. Due to similarities in clinical presentations and laboratory findings, it is difficult to differentiate between IPTs and neoplastic tumors, which can lead to misdiagnosis [80, 85]. However, IPT patients are often tested negative for alpha-fetoprotein, which may help distinguish IPT from hepatocellular carcinoma [79, 80, 85]. Cases of pseudotumor have been documented across a wide age range, from 6-month-old infants to 83-year-old adults, with a male preponderance [85, 86].

On imaging, IPT can mimic liver abscesses or malignant tumors, including peripheral intrahepatic cholangiocarcinoma and hepatocellular carcinoma [80]. Non-enhanced CT reveals hypoattenuating lesions, while contrast-enhanced CT demonstrates central hypoattenuating areas with a delayed hyperattenuating periphery [79]. Lymphoplasmacytic pseudotumor is usually located in the hilum and is distributed along hilar bile ducts, resembling hilar cholangiocarcinoma, while the fibrohistiocytic subtype is more often observed in liver parenchyma, mimicking intrahepatic cholangiocarcinoma. Nevertheless, most cases are still diagnosed by histological examinations following surgery.

As for laboratory results, IPT patients often have raised leukocytes, ESR, and CRP values [84, 87].

### Esophagus, stomach, and intestine involvement

Involvement of the esophagus, stomach and intestine is rare in IgG4-RD [66]. In the limited available literature, patients with IgG4-related esophageal disease usually present with dysphagia and gastroesophageal reflux-related symptoms [88]. Upon gastroscopy, the most prominent finding is esophageal stricture, and an esophageal mass has also been reported [88–90]. Stomach and intestine involvement is also rare and can manifest as diffuse wall thickening, gastric ulcer, gastric lesions, swollen major papilla, and polyps [89, 91, 92]. Patients may present with gastrointestinal symptoms such as anorexia, nausea, abdominal pain, weight loss, and anemia if they bleed from chronic mucosal ulceration [92, 93]. Rare case reports indicate that elevated IgG4 levels along with abnormal liver function tests may be observed, but more often laboratory results are unremarkable [93–96].

Diagnosis of IgG4-related esophagitis (IgG4-RE) is usually based on histopathology and immunohistochemistry, including a heightened number of IgG4-positive plasma cells, elevated IgG4:IgG ratio, characteristic pathological features, and exclusion of other autoimmune and proliferative diseases [58]. Gastrointestinal disease can be classified into two types. The first form is a gastrointestinal lesion characterized by marked wall thickening of the esophagus and stomach, featuring dense fibrosis and widespread infiltration of IgG4-positive plasma cells within the submucosa. The second is an IgG4-related pseudotumor, which presents as a polypoid or mass-like lesion in various locations, and can be detected by radiological examination [97]. Of note, infiltration of IgG4-positive plasma cells is frequently detected in the gastrointestinal tracts of patients with AIP and other autoimmune diseases, but these findings often lack additional histological features required for a definitive diagnosis of IgG4-RD [66, 97].

Recent evidence has established a statistical correlation between IgG4 antibodies and eosinophilic esophagitis (EoE), an antigen-mediated chronic disease characterized by dense mucosal eosinophilic infiltration. These two disease entities share many common features including tissue eosinophilia, fibrosis, and IgG4-positive cell infiltration [90, 98–100]. However, given the rarity of esophageal involvement in IgG4-RD, and with > 3000 eosinophils/µL serving as an exclusion criterion for IgG4-RD, EoE is still not considered part of the IgG4-RD spectrum [12]. Therefore, further research is needed to determine the exact role of IgG4 in EoE.

## Available treatments and long-term outcomes

Table 2 provides a practical summary of treatment options, relapse management, relapse risk stratification, and follow-up strategies across major gastroenterological manifestations.

**Table 2:** Practical management of major gastroenterological manifestations of IgG4-related disease

	AIP-1	IgG4-SC	Hepatic Involvement		Esophagus, Stomach, and Intestine Involvement
			IgG4-AIH	IPT	
Initial treatment	Prednisolone 0.6–1.0 mg/kg/day × 2–4 weeks, taper over ≥ 12 weeks; GC + DMARDs for high-risk patients	Prednisolone; > 80% achieve normalized ALP and radiological improvement	Prednisone alone or + azathioprine; biochemical response typically within 3 months	Conservative management preferred (corticosteroids, antibiotics, NSAIDs); surgery reserved for diagnostic uncertainty or failed conservative therapy	GC; esophageal dilation if stricture present; surgery if malignancy cannot be excluded
Relapse management	GC re-induction at individualized dose; early addition of DMARDs recommended; or B-cell targeting therapy (rituximab or inebilizumab)	GC re-induction + DMARDs / B-cell targeting therapy (rituximab or inebilizumab)	GC re-induction + azathioprine	Repeat conservative therapy; surgical intervention if refractory	GC re-induction; MMF for refractory cases; surgery if malignancy suspected
Relapse risk	High—56% relapse within 1 year, 92% by year 3; key risk factors: diffuse pancreatic enlargement, bile duct co-involvement, baseline IgG4 ≥ 6.5 g/L	High—relapse common after GC withdrawal; bile duct involvement and proximal strictures are key predictors	Moderate—80% maintain remission on azathioprine alone	Low—rarely progresses; spontaneous regression documented	Limited data—insufficient for formal risk stratification
Follow-up strategy	Initially every 1–3 months, then every 3–6 months after stabilization; Check complete blood count, liver function, total IgG, IgG4, complements; Check symptoms, IgG4-RD RI				
	CT/MRI or EUS for localized inflammation and structural assessment; progressive elevation of serum IgG4 as trigger for intensified monitoring	Serum IgG4, ALP, and bilirubin every 3–6 months; cholangiographic imaging if clinically indicated	Liver enzymes and serum IgG4 every 3–6 months; fibrosis-related imaging	Imaging (CT or MRI) to confirm mass regression	Endoscopic surveillance

### Treatment regimens

Although watchful waiting is suitable for asymptomatic cases, once vital organs are involved in IgG4-RD, aggressive treatment is imperative to prevent serious dysfunction or failure [101]. GCs are considered the first-line cornerstone treatment according to the international consensus [102]. The recommended initial therapy is 30–40 mg/day or 0.6 mg/kg/day of prednisone continued for 2–4 weeks, then tapered gradually with the goal of discontinuing glucocorticoids within 3 to 6 months from initiation of therapy. The daily dose may be reduced by 10 mg every two weeks until reaching 20 mg. Once stable on a 20-mg dose for a short period, the reduction can be changed to 5 mg every 2 weeks [102]. Patients receiving 0.6 mg/kg/day dosage of prednisolone, with reduction of 10% every 2 weeks and a maintenance dosage of 10 mg/day for a minimum of 3 months, had an overall response rate of 93.2% and 1-year relapse rate of 1.7% [103].

Although responsive to initial remission induction, patients often relapse after GC tapering, sometimes even when GCs are maintained at a high dosage [104]. In AIP, 56% of the patients relapse within 1 year and 92% relapse by year 3 of follow-up [105]. Different dosages of GCs resulted in similar remission rates, but a higher relapse rate was observed in the lower dosage group. Hence, patients with a greater extent of organ involvement and higher baseline disease severity may be at an increased risk of relapse, and may thus require higher glucocorticoid doses [103]. Furthermore, GCs are associated with significant adverse effects, such as diabetes mellitus, infections and osteonecrosis [105, 106]. Therefore, due to their toxicity and high disease recurrence, there remains a pressing need for GCs-sparing agents [9].

Opinions vary on whether steroid-sparing agents should be added for initial treatment, but gastroenterologists were least likely to include steroid-sparing agents in initial therapy [102]. Disease-modifying antirheumatic drugs (DMARDs) are often used in combination with GCs for induction therapy. Recent systematic review evidence demonstrated that GCs + DMARDs combination therapy had higher remission rates and lower relapse rates compared to GCs monotherapy [105]. The following medications are considered viable treatment options: azathioprine, mycophenolate mofetil, ciclosporin A, and leflunomide [107–109].

In cases of recurrent or refractory disease, B-cell depletion therapy represents a promising therapeutic strategy [110]. Inebilizumab, a humanized monoclonal antibody targeting CD19 + B cells, is the first and only therapy approved by the Food and Drug Administration for IgG4-RD. In the landmark MITIGATE trial—the first phase 3, multicenter, randomized, double-blind, placebo-controlled trial conducted in IgG4-RD—Inebilizumab reduced the risk of disease flares by 87% compared with placebo. In addition, Inebilizumab significantly improved flare-free, glucocorticoid-free complete remission rates, offering the potential to reduce long-term reliance on GCs [111, 112]. Rituximab, an anti-CD20 monoclonal antibody, has accumulated the most extensive real-world evidence among biologic agents for IgG4-RD, inducing a prompt and selective decline in serum IgG4 concentrations without significantly affecting other IgG subclasses, thereby enabling patients to achieve GCs’ independence [110, 113]. Exploratory studies suggest that other B-cell-targeting agents, such as Obexelimab and Telitacicept, may also hold promise, though further clinical validation is warranted [114, 115].

Beyond B-cell depletion, T-cell modulation and JAK/STAT pathway inhibition are being explored in IgG4-RD. Abatacept and Tofacitinib have demonstrated the ability to alleviate disease manifestations in IgG4-RD patients with digestive system involvement in a limited number of cases, though further validation is warranted [116, 117]. Additionally, a phase 2 trial investigating Filgotinib in refractory IgG4-RD is currently underway [118].

#### Type 1 autoimmune pancreatitis

Corticosteroids are the first-line therapy for patients with AIP-1. Guidelines recommend an initial prednisolone dose of 0.6–1.0 mg/kg/day for two to four weeks to induce remission, followed by gradual tapering over a minimum treatment duration of 12 weeks [11, 38]. According to a European cohort study [34], corticosteroids and rituximab demonstrated comparable complete remission rates in relapsing disease, achieving 78% and 73% respectively, suggesting no significant difference in therapeutic efficacy between these two treatment regimens.

#### IgG4-related sclerosing cholangitis

Corticosteroid therapy achieves excellent initial remission in IgG4-SC, with over 80% demonstrating normalized alkaline phosphatase levels and radiological improvement of biliary strictures [119–121]. However, relapse following treatment withdrawal is common, necessitating maintenance therapy and regular follow-up [122].

#### Hepatic involvement

Initial treatment with prednisone alone or in combination with azathioprine should be administered [70, 72, 123, 124]. Anti-inflammatory or immunosuppressive therapy is reported to be successful in the majority of cases. Most IgG4-AIH patients achieved biochemical response within 3 months [74]. This more rapid response distinguishes IgG4-AIH from classical AIH, for which the targeted benchmark is 6–12 months. Once remission is achieved, maintenance therapy with azathioprine alone is sufficient for 80% of the patients [125].

There are two contrasting approaches to managing IPT patients. The first strategy involves surgical resection, while the other favors conservative management. Surgery was the primary strategy in early literature and proved effective for the vast majority of patients, albeit with potentially life-threatening postoperative complications. However, conservative management is becoming increasingly popular, especially among patients who underwent diagnostic liver biopsy, since evidence suggests that IPT rarely progresses to malignancy [84, 87]. Since the 2000s, over half of the patients have received conservative therapy, including antibiotics, corticosteroids, and non-steroidal anti-inflammatory drugs [126]. This approach has resulted in complete or partial mass regression in most cases, although a subset has still required subsequent surgical intervention [80].

#### Esophageal, gastric, and intestinal involvement

Many patients with esophageal involvement received one or more esophageal dilations prior to diagnosis [88, 90]. Glucocorticoid therapy is generally effective, though relapsed cases may require additional mycophenolate mofetil or surgical intervention [88]. For gastric involvement, most patients underwent surgical resection, largely due to clinical suspicion of malignancy; such procedures could potentially be avoided if IgG4-RD was considered in the initial differential diagnosis [91, 96]. Given the rarity of isolated intestinal involvement, treatment data remain limited to case reports, and management generally follows the principles established for IgG4-RD overall.

### Relapse risk factors and predictors

IgG4-RD exhibits a relapse rate of 34–58% after glucocorticoid withdrawal [13], with multiple predictive factors identified through large-scale studies. A meta-analysis of 3797 patients demonstrated that history of allergy, multiorgan involvement, and decreased complement levels increase relapse risk [127]. Notably, central nervous system involvement confers particularly high risk (HR 21.13) [128], while bile duct involvement and diffuse pancreatic enlargement also predict relapse in IgG4-related pancreatitis [129]. Beyond clinical parameters, several laboratory markers independently predict relapse, including baseline serum IgG4 ≥ 6.5 g/L [130], failure to normalize at 6 months [128], total serum IgG ≥ 20.8 g/L, IgG4-RD Responder Index (RI) ≥ 9, and severe IgG4 + plasma cell infiltration in tissue specimens [130].

Treatment regimens significantly influence relapse outcomes. Initial prednisolone dosing of 0.4–0.69 mg/kg/day, with slow tapering at a rate of less than 0.4 mg/day and maintenance doses > 6.25 mg/day for over 3 years, reduces relapse risk [131, 132].

To translate these predictors into clinical practice, a three-variable prognostic score incorporating total serum IgG ≥ 20.8 g/L, IgG4-RD RI ≥ 9, and severe IgG4 + plasma cell infiltration demonstrated good discriminative performance (AUC 0.806); a score ≥ 2 predicted relapse with 83% sensitivity and 70% specificity, and 3-year relapse rates escalated from 0% (score 0) to 100% (score 3) [130]. For the minority of seronegative patients, a nomogram incorporating younger onset age, glucocorticoid monotherapy, elevated CRP, and elevated ESR has been proposed, with a baseline score greater than 84.65 identifying those at an elevated risk [133]. Although both tools require prospective validation, they provide a practical framework for bedside risk stratification.

### Management of relapse

For patients experiencing relapse after successful remission induction, glucocorticoid reinduction remains the cornerstone of retreatment [102]. However, unlike the standardized initial dosing of 0.6 mg/kg/day, reinduction doses at relapse are typically individualized. In a multicenter study, the mean peak glucocorticoid dose upon relapse was 21.6 ± 11.1 mg/day [132], and no consensus recommendations on relapse reinduction dosing currently exist.

A critical distinction from initial therapy is the recommended addition of steroid-sparing agents at relapse. International and Japanese guidelines recommend the early introduction of DMARDs during the maintenance phase following relapse, rather than continuing glucocorticoid monotherapy [58, 102, 134], given the substantially higher risk of rerelapse in this population.

For patients with multiple relapses or DMARDs-refractory disease, escalation to B-cell depletion therapy is warranted. Beyond its established role in refractory disease, rituximab administered as scheduled maintenance every 6 months achieved a 100% 18-month relapse-free rate, compared with 29% with on-demand retreatment alone [135]. Inebilizumab, as previously stated, represents the highest-level evidence for flare prevention in this setting [112].

### Long-term outcomes and surveillance strategies

Given the subtle nature of relapse, including clinical and serological relapse, serial assessments are essential. The monitoring strategy comprises continuous serum IgG4 tracking to guide preemptive therapy, regular IgG4-RD RI calculation to quantify disease activity, and imaging (CT, MRI, or endoscopic ultrasound) to evaluate structural organ changes. Guidelines emphasize comprehensive evaluation over isolated IgG4 reliance [12, 102, 136], as elevations alone sometimes correlate poorly with disease activity. However, persistent elevation of serum IgG4 should not be ignored; empiric treatment of a rising serum IgG4 may be warranted for patients in whom relapse may lead to severe consequences.

During early follow-up after remission induction, comprehensive assessments are recommended every 3–6 months, even in the absence of overt symptoms, as IgG4-RD is prone to subclinical progression that may lead to irreversible organ fibrosis [137]. Once disease stability is confirmed over a sustained period, follow-up intervals may be gradually extended. High-risk patients, particularly those with multiorgan involvement, elevated baseline IgG4, or a history of prior relapse, warrant intensified monitoring schedules. For seropositive patients under surveillance, an IgG4-Ratio greater than 1.6 between consecutive measurements—reflecting a rise of more than 60% at approximately 3-month intervals—has been identified as a threshold predictive of impending relapse [138]. Nonetheless, no prospective studies have yet directly compared intensified versus standard follow-up schedules in high-risk individuals; the optimal monitoring frequency and imaging interval in this population remain to be defined by future research.

Furthermore, long-term management should not neglect the chronic toxicities of corticosteroids and immunosuppressants, such as osteoporosis, diabetes, and opportunistic infections, to minimize iatrogenic complications. Proactive monitoring for treatment-related adverse effects, including bone density assessment and metabolic screening, should be integrated into routine follow-up protocols.

## Conclusions

Gastrointestinal involvement is one of the most common clinical manifestations in IgG4-RD. The convergence of genetic, environmental, and immunological evidence provides a coherent framework for understanding its clinical heterogeneity. While glucocorticoids remain the mainstay of therapy and achieve high initial response rates, the persistently high relapse rates and cumulative steroid toxicity represent an unmet clinical need. This gap is now beginning to be addressed by the recent approval of inebilizumab and upcoming clinical trials. Optimal treatment duration and tapering protocols will be established through prospective, controlled studies.
